# Impact of Diet Composition in Adult Offspring is Dependent on Maternal Diet during Pregnancy and Lactation in Rats

**DOI:** 10.3390/nu8010046

**Published:** 2016-01-14

**Authors:** Megan C. Hallam, Raylene A. Reimer

**Affiliations:** 1 Faculty of Kinesiology, University of Calgary, 2500 University Drive NW, Calgary, AB T2N 1N4, Canada; hallammc@gmail.com; 2 Department of Biochemistry & Molecular Biology, University of Calgary, 3330 Hospital Drive NW, Calgary, AB T2N 4N1, Canada

**Keywords:** maternal diet, prebiotics, dietary protein, fetal programming, obesity

## Abstract

The Thrifty Phenotype Hypothesis proposes that the fetus takes cues from the maternal environment to predict its postnatal environment. A mismatch between the predicted and actual environments precipitates an increased risk of chronic disease. Our objective was to determine if, following a high fat, high sucrose (HFS) diet challenge in adulthood, re-matching offspring to their maternal gestational diet would improve metabolic health more so than if there was no previous exposure to that diet. Animals re-matched to a high prebiotic fiber diet (HF) had lower body weight and adiposity than animals re-matched to a high protein (HP) or control (C) diet and also had increased levels of the satiety hormones GLP-1 and PYY (*p* < 0.05). Control animals, whether maintained throughout the study on AIN-93M, or continued on HFS rather than reverting back to AIN-93M, did not differ from each other in body weight or adiposity. Overall, the HF diet was associated with the most beneficial metabolic phenotype (body fat, glucose control, satiety hormones). The HP diet, as per our previous work, had detrimental effects on body weight and adiposity. Findings in control rats suggest that the obesogenic potential of the powdered AIN-93 diet warrants investigation.

## 1. Introduction

While *in utero* the fetus is subjected to numerous cues about the environment it will encounter in postnatal life. The fetus is completely dependent on maternal nutrient supply for normal growth and development and therefore many of the environmental cues it encounters are linked to maternal diet. When a mismatch occurs between the nutritional environment predicted by the fetus and the actual environment encountered postnatally, the risk of developing chronic diseases, such as metabolic syndrome or type 2 diabetes, hypertension or cardiovascular disease, is increased [1]. More recent evidence also shows detrimental effects on appetite regulation and glucose and lipid metabolism in response to nutritional mismatch [2]. This programming effect has often been examined in terms of an adverse maternal environment, such as energy or protein restriction, drug treatment, or a maternal high fat diet which is then mismatched in offspring with provision of a control diet or a high energy density diet, either at weaning or in adulthood. Even small degrees of mismatch can have negative consequences on offspring, although in general the greater the mismatch the greater the consequences [3]. While it is typical for a weaning diet high in fat to have negative effects on offspring health regardless of maternal diet, there are some metabolic markers that have been shown to improve with consistent dietary exposure from the pre- to post-natal period. Offspring of high fat-diet fed dams who were also fed high fat diet at weaning had decreased plasma triglycerides and improved endothelial function compared to their littermates given a control diet [3]. This finding highlights the importance of the predictive adaptive response and provides justification for examining not only detrimental patterns of pre- and postnatal nutrition but also whether a healthy maternal diet, matched to offspring postnatal diet could in fact result in the lowest disease risk.

A diet high in prebiotic fiber has been shown to have beneficial effects on satiety, food intake, triglyceride accumulation and glycemic control [4,5,6,7]. We have previously shown that prenatal exposure via a maternal diet high in prebiotic fiber (HF) results in decreased weight gain and body fat in the offspring when they are challenged with a high fat, high sucrose diet (HFS) in adulthood [8]. With postnatal exposure, wherein rat pups were weaned onto a high prebiotic diet, challenged in adulthood with the HFS diet and then re-matched to the prebiotic diet, reduced adiposity was observed compared to animals re-matched to a high protein (HP) diet [7]. In contrast to maternal diets high in prebiotic fiber, maternal diets high in protein have been shown to predispose offspring to excessive weight gain and adiposity when challenged with HFS in adulthood [8]. Similar detrimental effects on body weight also occurred with exposure to a HP diet at weaning and throughout growth followed by a HFS diet in adulthood [9]. However, when rats that were weaned onto a HP diet were once again allowed to consume a HP diet after transient HFS exposure, glycemic response, as well as percent body fat was normalized, showing potential value in re-matching to a nutritional environment encountered early in life [7]. While the study showed that re-matching to an early post-natal diet had metabolic benefits, it is not known if re-matching offspring to their maternal pre-natal HP diet after a dietary HFS challenge in adulthood could also have beneficial effects on the offspring.

The purpose of this study was to examine the impact of dietary patterns represented by mismatching and re-matching between prenatal and adulthood diets in rats. Our first aim was to evaluate the impact of re-matching offspring in adulthood to their maternal gestational diets high in prebiotic fiber or protein after a HFS diet challenge. The secondary aim was to provide important reference data with regards to normal growth and metabolic response to consumption of the control AIN-93 diet throughout life or continuous HFS diet consumption from 14 to 28 weeks of age (*i.e.*, no re-matching to maternal diet).

## 2. Materials and Methods

### 2.1. Ethical Approval

The University of Calgary Animal Care Committee approved the experimental protocol which was conducted in accordance with the *Guide for the Care and Use of Laboratory Animals*.

### 2.2. Animals and Diets

Thirty-seven virgin Wistar dams were obtained from Charles River (Montreal, QC, Canada) at 13 weeks of age and acclimatized for 1 week in a temperature and humidity controlled facility with a 12-h light/dark cycle prior to starting the intervention at 14 weeks of age. Animals were given one of three nutritionally complete experimental diets: high prebiotic fiber (HF) (21.6% wt/wt, 1:1 ratio of oligofructose and inulin; 13.73 kJ/g), high protein (HP) (40% wt/wt; 15.74 kJ/g), or control (C) (based on AIN-93G; 15.74 kJ/g). All maternal diets were mixed in house using ingredients purchased from Dyets, Inc. (Bethlehem, PA, USA); the detailed composition has been previously published [10]. An additional 15 dams consumed AIN-93G throughout pregnancy and lactation to form a reference group. Dams consumed the diets for one week prior to being bred with male Wistar rats in wire-bottomed cages. Fourteen week old male Wistar rats were purchased from Charles River (Montreal, QC, Canada) and consumed standard rat chow (Lab Diet #5001, Lab Diet, St. Louis, MO, USA). Following the identification of a copulation plug, dams were housed individually and continued to consume their assigned experimental diet (C, HF, or HP) until the pups were weaned. Dams were weighed weekly, and food intake was measured daily throughout week 2 of pregnancy.

**Figure 1:**
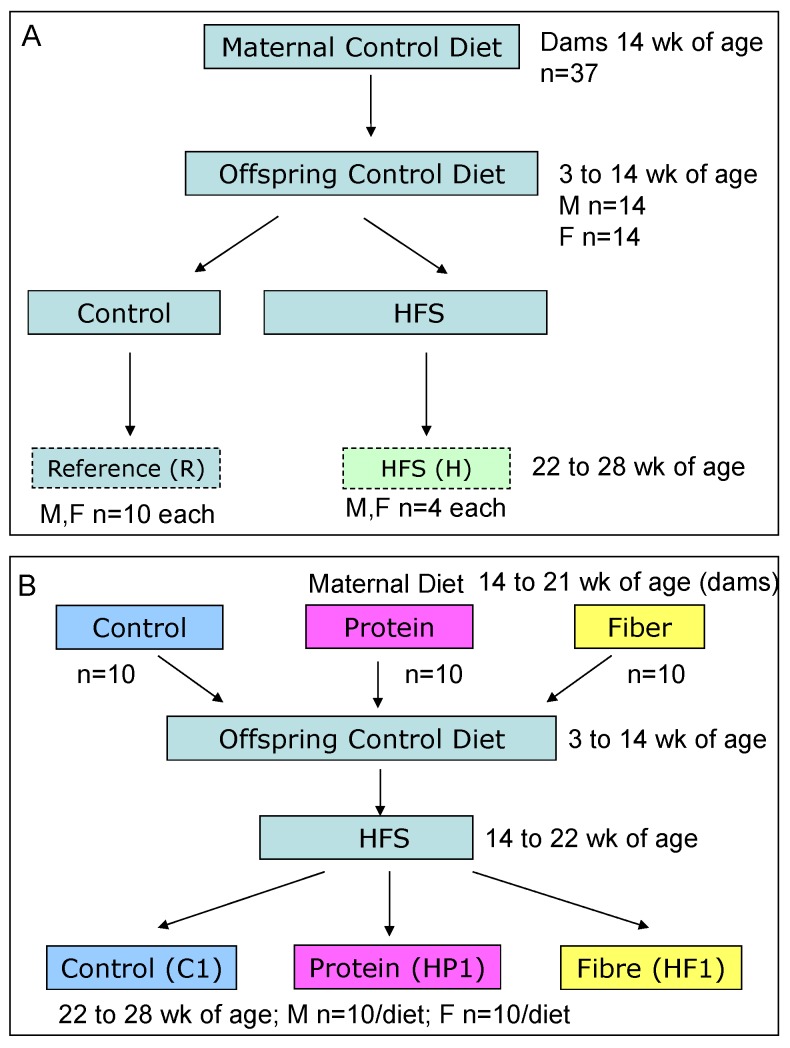
Schematic of study design for Reference (**A**) and Experimental (**B**) groups.

Pups were weighed on the day after birth, and litters then culled to 10 pups with equal numbers of males and females where possible. Offspring were weighed weekly for the remainder of the study. Food intake was measured for 5 consecutive days out of every 20 days by subtracting the weight of the cup and diet from the previous days’ weight. At weaning (3 weeks), 1 male and 1 female pup were randomly selected from each litter to continue in the study until 28 weeks of age. By selecting one male and one female from each litter we examined *n* = 10 individual rats per sex that were each derived from a different litter, minimizing the effect of any single dam. Therefore, the total number of offspring is *n* = 10 males and *n* = 10 females, each from different dams who consumed either control (*n* = 10), high protein (*n* = 10) or high fiber (*n* = 10). Pups were weaned onto AIN-93G control diet [11]. Offspring were then switched to AIN-93M (15.07 kJ/g) for maintenance at 10 weeks of age. At 14 weeks of age, offspring were fed a high fat, high sucrose (HFS) diet (19.26 kJ/g) for 8 weeks (Table 1). Thirteen males and 13 females were kept on AIN-93M as a reference group (R). This reference group, matched for age and sex to the intervention groups, provides a standard of normal growth in these rats. After 8 weeks on HFS, rats were re-matched to the diet of their respective dams for 6 weeks and thereafter identified as C1, HP1 or HF1 (Table 1). Four males and females continued to consume the HFS diet for the final 6 weeks (H). The overall study design is depicted in Figure 1.

**Table 1:** Maintenance and HFS Diet Compositions.

Ingredient (g/kg)	Control (AIN-93M)	High Protein	High Fiber	HFS
Cornstarch	465.7	205.7	325.5	49.5
Casein	140.0	400.0	120.4	140.0
Dyetrose	155.0	155.0	133.0	-
Sucrose	100.0	100.0	86.0	510.0
Soybean Oil	40.0	40.0	34.4	100.0
Lard	-	-	-	100.0
Alphacel	50.0	50.0	43.0	50.0
AIN-93M Mineral Mix	35.0	35.0	30.1	35.0
AIN-93-VX Vitamin Mix	10.0	10.0	8.6	10.0
l-Cystine	1.8	1.8	1.6	2.0
dl-Methionine	-	-	-	3.0
Choline Bitartrate	2.5	2.5	2.2	2.5
Inulin	-	-	107.6	-
Oligofructose	-	-	107.6	-

HFS, High fat, high sucrose. Inulin supplied as Orafti Raftiline HP and oligofructose as Orafti Raftilose P95 in a 1:1 blend by weight (Quadra Chemicals Ltd., Burlington, ON, Canada). The energy value of the fiber blend (1.5 kcal/g) was used to substitute for an equicaloric amount of cornstarch in the high fiber diet.

### 2.3. Oral Glucose Tolerance Test and Tissue Sampling

Four days before the end of the study, rats were fasted overnight and an oral glucose tolerance test (OGTT) performed. Blood was sampled from the tip of the tail in conscious rats followed by an oral glucose gavage (2 g/kg). At 15, 30, 60 and 90 min post-glucose gavage, additional blood was sampled from the tail and immediately analyzed using a blood glucose meter (Accu-Chek Blood Glucose Meter, Laval, QC, Canada). One day prior to study termination rats underwent a DXA scan (Hologic ODR 4500; Hologic Inc., Bedford, MA, USA) while lightly anesthetized using isoflurane. Hologic QDR software for small animals was used to determine lean mass, fat mass and bone mineral density. A second OGTT for satiety hormone analysis was performed at the time of terminal tissue collection and blood sampled at 0, 15, 30, 60 and 90 min into tubes containing diprotinin-A, Sigma protease inhibitor and Roche Pefabloc at concentrations previously described [12]. Plasma was stored at −80 °C until analysis. The OGTT was a terminal procedure and after the 90 min blood collection rats were killed via over-anesthetization and aortic cut. The stomach, small intestine, cecum, colon and liver were measured and a sample of each was snap frozen in liquid nitrogen and stored at −80 °C. 

### 2.4. Plasma Analysis

A Milliplex Rat Gut Hormone kit (Millipore, St. Charles, MO, USA) and Luminex instrument (Eve Technologies, Calgary, AB, Canada) were used to measure ghrelin (active), insulin, amylin (active), leptin, glucose-dependent insulinotropic polypeptide (GIP) (total) and peptide tyrosine tyrosine (PYY) (total). This assay has intra-assay precision of 1%–8% and inter-assay precision of 7%–24%. An ELISA was used to measure active GLP-1 (Millipore). Intra-assay precision is reported to be 8% ± 4.8% and inter-assay precision is reported to be 7.4% ± 1.1%. Homeostasis model assessment of insulin resistance (HOMA-IR) was calculated from fasting insulin and fasting glucose.

### 2.5. Hepatic Triglyceride Analysis

Triglyceride content of the liver was quantified using 25 mg of tissue according to the manufacturer guidelines of the GPO reagent set (Pointe Scientific Inc., Lincoln Park, MI, USA).

### 2.6. RNA Extraction and Real-Time PCR

Total RNA was extracted from the liver using TRIzol reagent (Invitrogen, Carlsbad, CA, USA). Reverse transcription was performed with an input of 1 μg of total RNA using the 1st strand cDNA synthesis kit for RT-PCR (Invitrogen) with oligo d(T)15 as a primer. The cDNA was amplified using primers synthesized by the University of Calgary Core DNA Services (Calgary, AB, Canada) and analyzed by real time PCR. Primer sequences for Acetyl Co-A Carboxylase (ACC), Fatty Acid Synthase (FAS), sterol regulatory element binding protein-1c (SREBP1c), glucose-6-phosphatase and AMP-activated protein kinase alpha-1 (AMPKα1) were according to our previous work [10]. A melt curve showed the melting point of the PCR product of interest. Glyceraldehyde-3-phosphate dehydrogenase (GAPDH) was verified as a suitable housekeeping gene for the tissues of interest and GAPDH primers included as an internal control in the reactions. The 2^−∆CT^ method (ΔCT = CT (gene of interest)—CT (reference gene)) was utilized for the data analysis where threshold cycle (CT) indicates the fractional cycle number at which the amount of amplified target reaches a fixed threshold [13]. The ∆CT is the difference in threshold cycles for the gene of interest and GAPDH.

### 2.7. Statistical Analysis

All data are presented as mean ± SEM. Data collected from the dams was analyzed with one-way ANOVA with Tukey’s *post hoc* analysis. In offspring, a two-way ANOVA was used to compare the main effects of diet and sex and their interaction. Only when a significant interaction effect was identified were all groups, as applicable, compared using a one-way ANOVA with Tukey’s *post hoc* analysis. *p* ≤ 0.05 was considered significant. Statistical analysis was performed using SPSS v 19.0 software (Chicago, IL, USA).

## 3. Results

### 3.1. Offspring Re-Matched to Maternal HP, HF or C

The following results describe the outcomes in rats re-matched to the diet they were exposed to *in utero*, either control AIN-93 (C1), HF (HF1), or HP (HP1).

#### 3.1.1. Growth and Energy Intake

**Figure 2:**
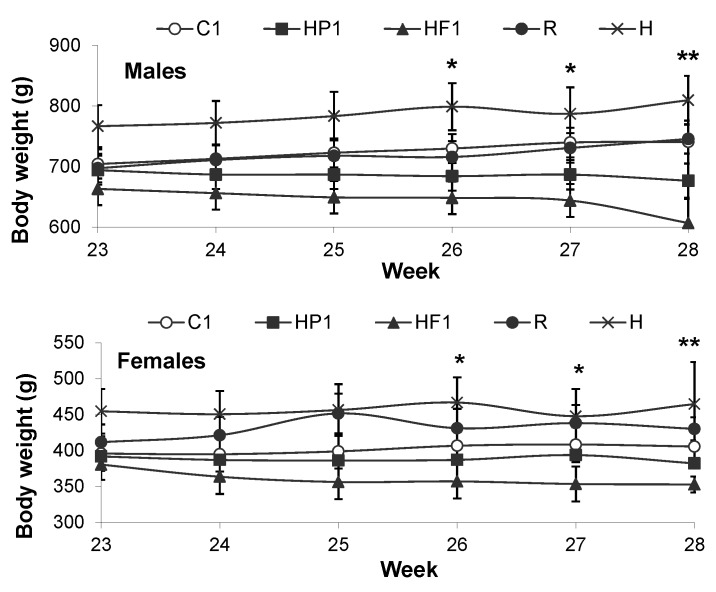

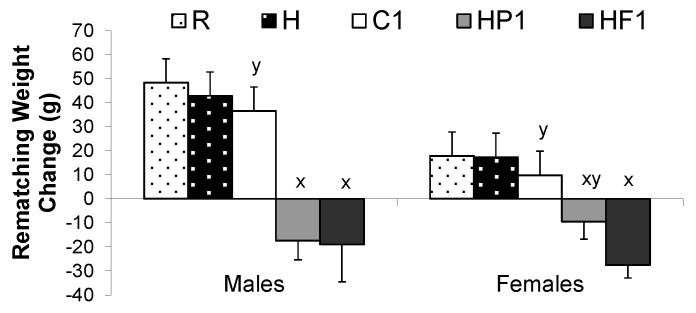
Body weight and weight gain of rats over the six week re-matching period following an eight week period of high fat, high sucrose diet consumption. Values are means ± SEM, *n* = 10 per group except *n* = 4 for group H. * represents a significant (*p* < 0.05) difference between HF1 and C1. ** represents a significant (*p* < 0.05) difference within HF1 between 23 and 28 weeks. For weight change, treatments with different letters are significantly different between diets (*p* < 0.05): superscripts ^x,y^ indicate significant difference between C1, HP1, HF1. There were no differences between R, H and C1.

The growth trajectory of the pups to the end of the HFS period (22 weeks) has been previously reported [8]. Among animals being re-matched to the HF, HP or C diets, body weight was significantly affected by the interaction of time and diet (*p* < 0.001) (Figure 2). At weeks 26 and 27, HF1 rats weighed less than C1 rats for both males and females. When re-matched to their maternal diet following the HFS challenge (22–28 weeks), HF1 rats lost weight resulting in a final body weight that was significantly lower than that at week 23. There was also an independent effect of sex with males weighing more than females (*p* < 0.001). When expressed as change in weight from 23 to 28 weeks, male HP1 and HF1 lost weight and C1 gained weight while in females only the weight loss in HF1 offspring was significantly different from the weight gain in C1.

**Figure 3:**
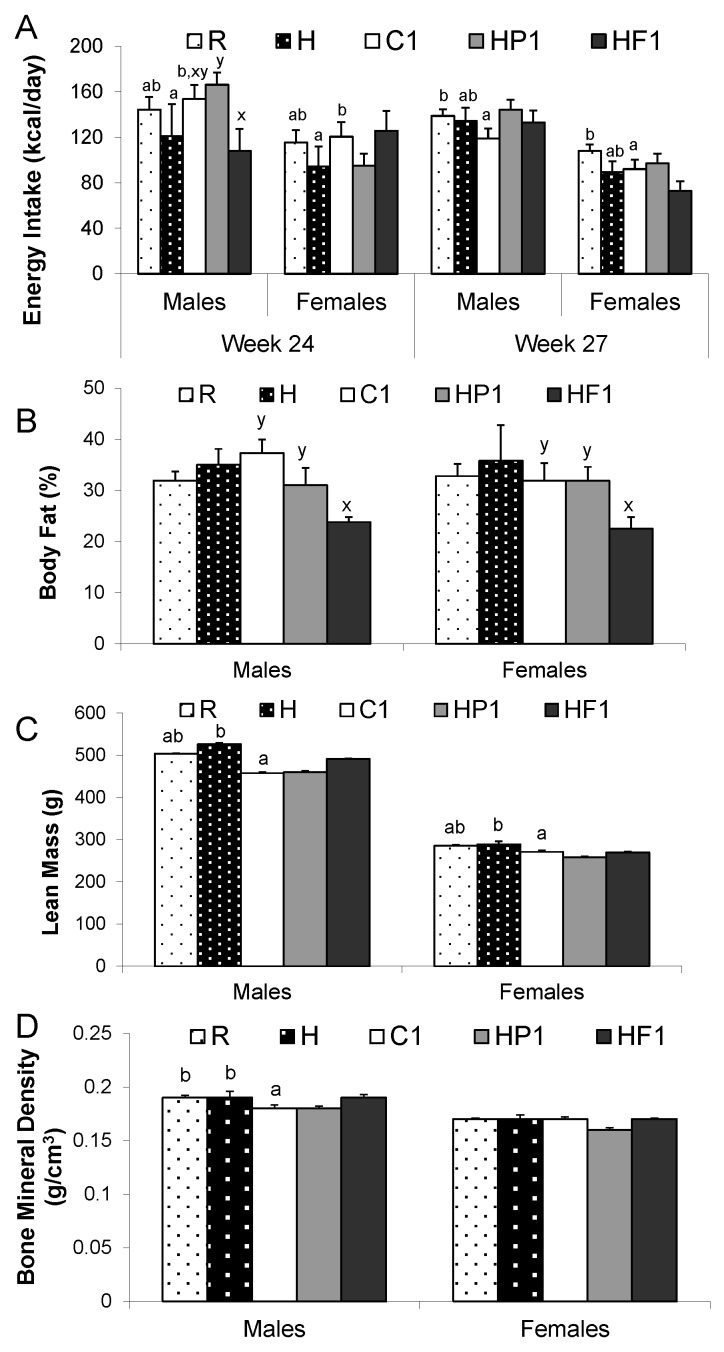
Energy intake (**A**), percent body fat (**B**), lean mass (**C**) and bone mineral density (**D**) at weeks 24 and 27 for all groups. Values are means ± SEM, *n* = 10 per group except *n* = 4 for group H. Treatments with different letters are significantly different between diets: superscripts ^x,y^ indicate significant difference between C1, HP1, HF1; superscripts ^a,b^ indicate significant difference between R, H and C1 (*p* < 0.05).

Energy intake was affected by the interaction of diet and sex at 24 weeks of age with male HP1 consuming more energy than male HF1 (*p* = 0.034) (Figure 3). At 27 weeks there was a significant sex effect with males consuming more energy than females (*p* < 0.001).

#### 3.1.2. Body Composition

Male and female HF1 rats had lower body weight and percent body fat than C1 and HP1 rats at 28 weeks of age (*p* = 0.009) (Figure 3). Small intestine length, colon length and weight, and cecum weight were greater in HF1 than C1 and HP1 rats at euthanization at 28 weeks of age (*p* < 0.001) (Table 2). Kidney weight was greater in HP1 than HF1 and C1 (*p* = 0.037).

**Table 2:** Physical characteristics of offspring at 28 weeks.

		Diet Group				
	Sex	HP1	HF1	C1	R	H
Liver (g)	M	16.1 ± 0.5	15.5 ± 0.7	17.3 ± 0.7	18.8 ± 0.9	19.0 ± 1.0
	F	9.5 ± 0.6	8.9 ± 0.4	9.8 ± 0.8	9.0 ± 1.2	10.2 ± 1.1
Stomach (g)	M	2.7 ± 0.1	2.6 ± 0.1	2.8 ± 0.1 ^a^	3.3 ± 0.1 ^b^	3.1 ± 0.2 ^b^
	F	2.0 ± 0.1	2.0 ± 0.1	1.9 ± 0.1 ^a^	1.9 ± 0.2 ^b^	2.4 ± 0.3 ^b^
Small Intestine Length (cm)	M	130.5 ± 1.5 ^x^	143.8 ± 2.7 ^y^	125.7 ± 2.4 ^x,a^	134.8 ± 2.2 ^b^	133.8 ± 1.3 ^a,b^
	F	115.0 ± 3.4 ^x^	122.8 ± 2.5 ^y^	116.8 ± 3.7 ^x,a^	104.6 ± 2.2 ^b^	122 ± 5.5 ^a,b^
Small Intestine Weight (g)	M	8.1 ± 0.4	8.7 ± 0.4	7.9 ± 0.2	8.2 ± 0.24	8.6 ± 0.13
	F	6.1 ± 0.4	6.1 ± 0.4	6.8 ± 0.3	5.4 ± 0.64	6.3 ± 0.41
Cecum (g)	M	1.07 ± 0.1 ^x^	3.2 ± 0.3 ^y^	1.01 ± 0.1 ^x,a^	1.5 ± 0.04 ^b^	0.63 ± 0.36 ^a^
	F	0.74 ± 0.06 ^x^	2.3 ± 0.3 ^y^	0.8 ± 0.1 ^x,a^	1.0 ± 0.19 ^b^	0.88 ± 0.29 ^a^
Colon Length (cm)	M	23.5 ± 0.7 ^x^	26.1 ± 0.9 ^y^	21.6 ± 0.7 ^x^	21.1 ± 0.43	23.3 ± 0.85
	F	19.6 ± 0.3 ^x^	24.8 ± 0.4 ^y^	20.0 ± 0.7 ^x^	16.4 ± 1.9	20.5 ± 1.3
Colon Weight (g)	M	2.1 ± 0.1 ^y^	2.4 ± 0.2 ^z^	1.8 ± 0.1 ^x^	1.6 ± 0.05	1.9 ± 0.1
	F	1.5 ± 0.1 ^y^	2.0 ± 0.1 ^z^	1.3 ± 0.05 ^x^	1.1 ± 0.13	1.3 ± 0.06
Kidneys (g)	M	4.0 ± 0.1 ^y^	3.4 ± 0.1 ^x^	3.6 ± 0.1 ^x^	3.5 ± 0.1	3.8 ± 0.2
	F	2.4 ± 0.1 ^y^	2.1 ± 0.1 ^x^	2.3 ± 0.1 ^x^	1.9 ± 0.2	2.4 ± 0.3
Nasoanal Length (cm)	M	28.9 ± 0.16	28.6 ± 0.34	28.5 ± 0.19	28.4 ± 0.16	28.8 ± 0.32
	F	24.0 ± 0.41	23.5 ± 0.19	24.0 ± 0.17	23.6 ± 0.27	23.8 ± 0.32

HP1, offspring of high protein (HP) dams re-matched to HP in adulthood; HF1, offspring of high prebiotic fiber (HF) dams re-matched to HF in adulthood; C1, offspring of control dams re-matched to control in adulthood; R, reference group no high fat/sucrose (HFS) consumed; H, continued on HFS after other groups re-matched; M, male; F, female. Values are means ± SEM, *n* = 10 per group except *n* = 4 for group H. Values with different letters are significantly different from each other: superscripts ^x,y,z^ indicate significant difference between C1, HP1, HF1; superscripts ^a,b^ indicate significant difference between R, H and C1 (*p* < 0.05).

#### 3.1.3. Plasma Satiety Hormones and Blood Glucose

In males, fasting insulin was lower in HF1 and HP1 compared to C1 (Table 3). In males and females, fasting PYY and GLP-1 were higher in HF1 *versus* C1 and HP1 while leptin was lower in HF1 compared to C1 and HP1 (*p* < 0.05). The interaction between diet and sex affected HOMA-IR scores, which were lower in male HF1 and HP1 than male C1 rats (*p* = 0.012) (Table 3).

**Table 3:** Fasting blood glucose, plasma satiety hormones, HOMA-IR and liver triglycerides in adult offspring re-matched to maternal diet.

		Diet Group				
	Sex	HP1	HF1	C1	R	H
Glucose (mmol/l)	M	6.38 ± 0.29	5.59 ± 0.32	5.65 ± 0.28 ^b^	4.98 ± 0.08 ^a^	4.88 ± 0.45 ^a^
	F	6.16 ± 0.54	5.88 ± 0.45	5.83 ± 0.34 ^b^	3.20 ± 0.67 ^a^	4.55 ± 0.17 ^a^
Insulin (pg/mL)	M	2565 ± 443 ^x^	3312 ± 633 ^x^	5958 ± 731 ^y,b^	3022 ± 595 ^a^	1888 ± 150 ^a^
	F	1880 ± 274	1321 ± 343	1692 ± 513 ^a^	1599 ± 394 ^a,b^	2375 ± 1133 ^a,b^
Amylin (pg/mL)	M	56.4 ± 10.5	63.0 ± 5.7	75.0 ± 11.1 ^a^	43.1 ± 6.8 ^a^	418.6 ± 145.2 ^b^
	F	52.8 ± 9.4	54.3 ± 4.8	52.2 ± 8.1 ^a,b^	37.3 ± 6.4 ^a,b^	51.5 ± 9.7 ^a^
Ghrelin (ng/mL)	M	159.4 ± 36.8	235.1 ± 39.2	233.1 ± 39.7	266.8 ± 52.1	171.4 ± 32.1
	F	246.1 ± 50.7	369.6 ± 47.7	344.7 ± 55.4	529.1 ± 74.4	402.7 ± 65.7
GIP (ng/mL)	M	46.5 ± 8.3	47.8 ± 6.6	49.9 ± 6.3	49.9 ± 6.3	87.3 ± 14.0
	F	33.4 ± 9.2	42.1 ± 3.2	41.6 ± 8.4	49.7 ± 5.3	34.9 ± 6.1
PYY (pg/mL)	M	62.7 ± 7.5 ^x^	262.5 ± 43.2 ^y^	64.1 ± 4.4 ^x^	72.4 ± 6.3	54.0 ± 10.9
	F	63.5 ± 8.9 ^x^	201.3 ± 35.1 ^y^	55.8 ± 5.0 ^x^	62.2 ± 13.6	36.1 ± 4.9
GLP-1 (pg/mL)	M	6.0 ± 0.58 ^x^	8.4 ± 0.79 ^y^	7.22 ± 0.48 ^x,a,b^	6.5 ± 0.42 ^a,b^	5.4 ± 0.19 ^a,b^
	F	6.36 ± 0.37 ^x^	8.54 ± 1.5 ^y^	4.45 ± 0.72 ^x,a^	6.4 ± 0.37 ^b^	4.82 ± 0.35 ^a,b^
Leptin (ng/mL)	M	21.0 ± 4.2 ^y^	14.5 ± 2.2 ^x^	27.2 ± 2.5 ^y^	20.4 ± 1.9	18.4 ± 2.4
	F	10.3 ± 1.6 ^y^	6.5 ± 1.0 ^x^	11.1 ± 2.1 ^y^	12.9 ± 1.4	11.3 ± 4.1
HOMA-IR	M	20.2 ± 3.8 ^x^	19.0 ± 3.4 ^x^	35.9 ± 4.8 ^y,b^	15.3 ± 2.9 ^a^	9.8 ± 1.1 ^a^
	F	10.4 ± 1.6 ^z^	5.8 ± 1.1 ^z^	7.1 ± 1.5 ^z,d^	8.8 ± 2.2 ^a,b,c,d^	35.5 ± 4.5 ^c^
Liver TG (mg/mg protein)	M	36.6 ± 3.1 ^y^	30.8 ± 3.1 ^x^	43.1 ± 3.7 ^y^	33.1 ± 2.5	30.4 ± 4.0
	F	26.8 ± 2.5 ^y^	23.2 ± 1.5 ^x^	32.2 ± 1.6 ^y^	36.8 ± 2.0	35.5 ± 4.5

HP1, offspring of high protein (HP) dams re-matched to HP in adulthood; HF1, offspring of high prebiotic fiber (HF) dams re-matched to HF in adulthood; C1, offspring of control dams re-matched to control in adulthood; R, reference group no high fat/sucrose (HFS) consumed; H, continued on HFS after other groups re-matched; M, male; F, female. GIP, glucose-dependent insulinotropic polypeptide; PYY, peptide tyrosine tyrosine; GLP-1, glucagon-like peptide-1; HOMA-IR, homeostasis model assessment of insulin resistance; TG, triglycerides. Values are means ± SEM, *n* = 10 per group except *n* = 4 for group H. Values with different letters are significantly different from each other: superscripts ^x,y,z^ indicate significant difference between C1, HP1, HF1; superscripts ^a,b,c,d^ indicate significant difference between R, H and C1 (*p* < 0.05).

**Figure 4:**
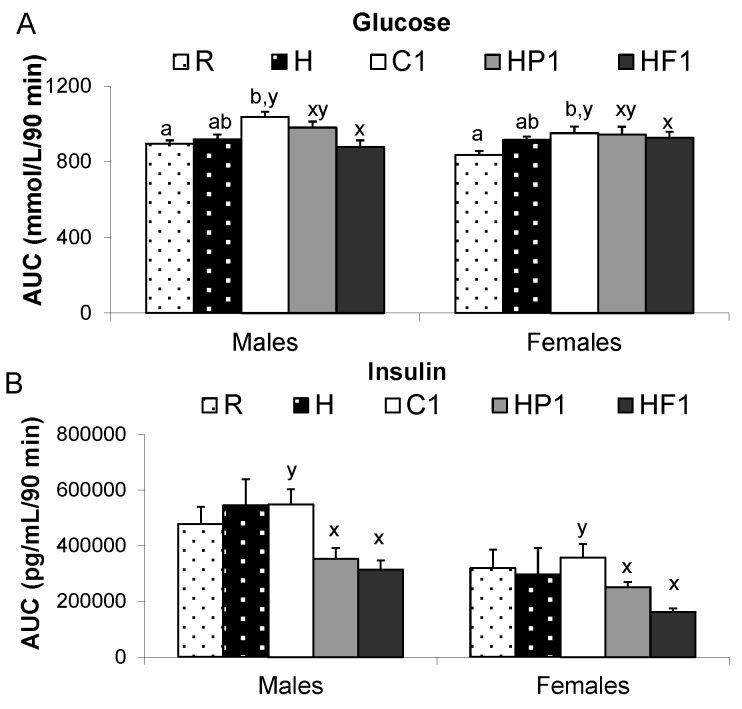

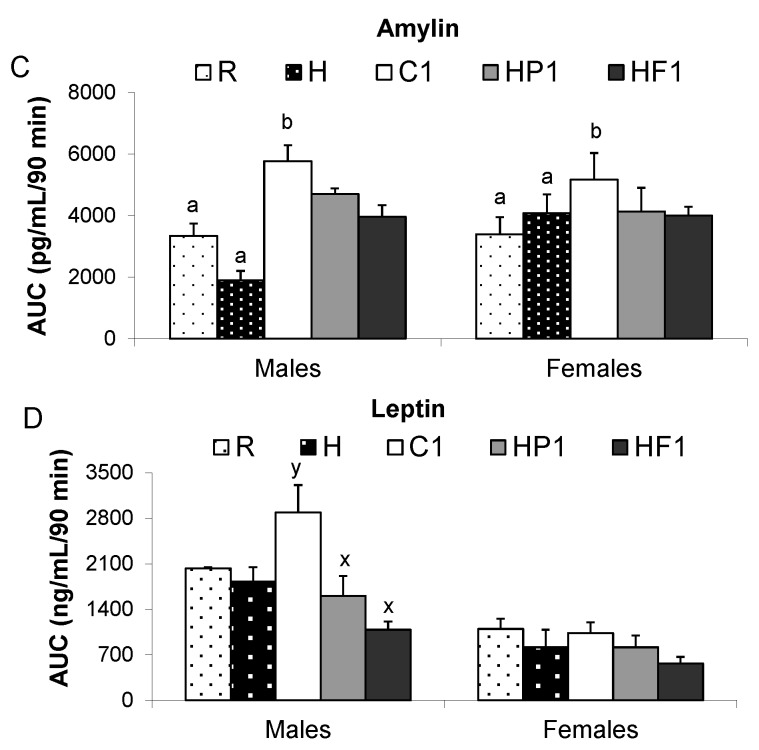
Area under the curve for glucose (**A**), insulin (**B**), amylin (**C**) and leptin (**D**) over a 90 min oral glucose tolerance test (OGTT) at 28 weeks. Values are means ± SEM, *n* = 10 per group except *n* = 4 for group H. Treatments with different letters are significantly different between diets: superscripts ^x,y^ indicate significant difference between C1, HP1, HF1; superscripts ^a,b^ indicate significant difference between R, H and C1 (*p* < 0.05).

**Figure 5:**
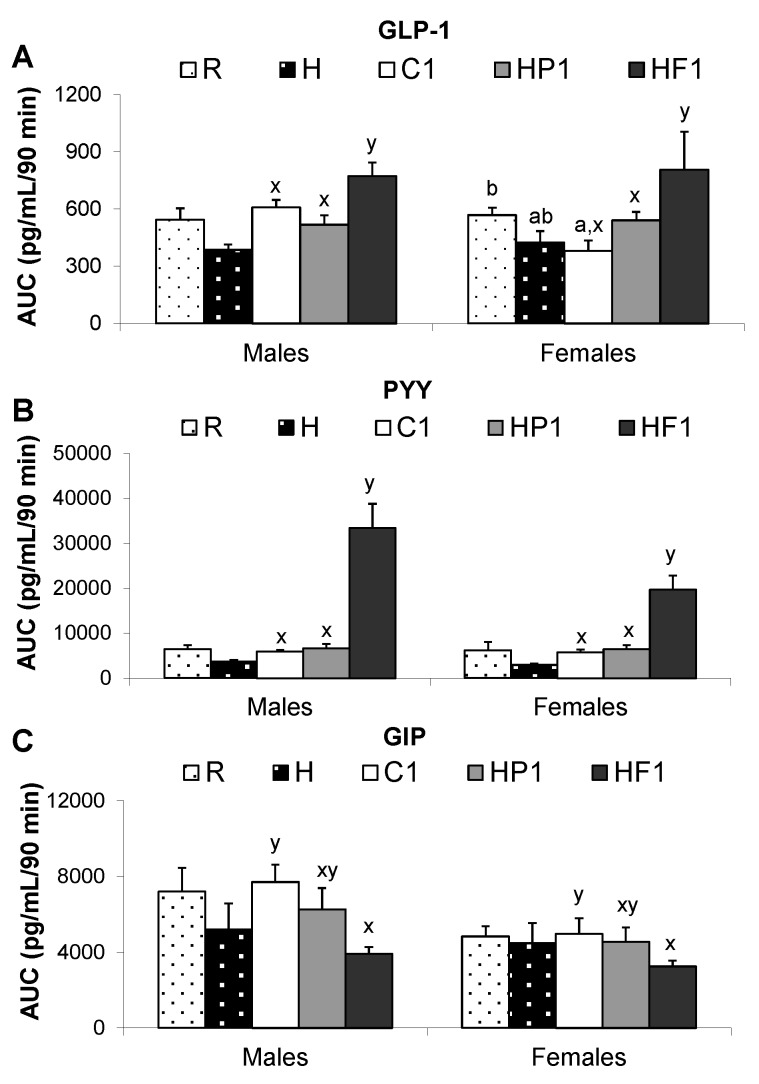

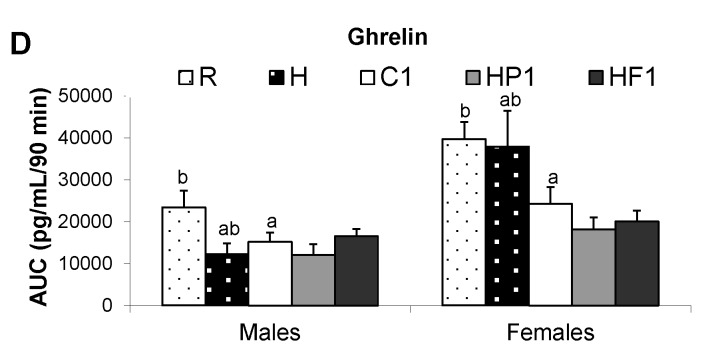
Area under the curve for GLP-1 (**A**), PYY (**B**), GIP (**C**) and ghrelin (**D**) over a 90 min OGTT at 28 weeks. Values are means ± SEM, *n* = 10 per group except *n* = 4 for group H. Treatments with different letters are significantly different between diets: superscripts ^x,y^ indicate significant difference between C1, HP1, HF1; superscripts ^a,b^ indicate significant difference between R, H and C1 (*p* < 0.05).

**Figure 6:**
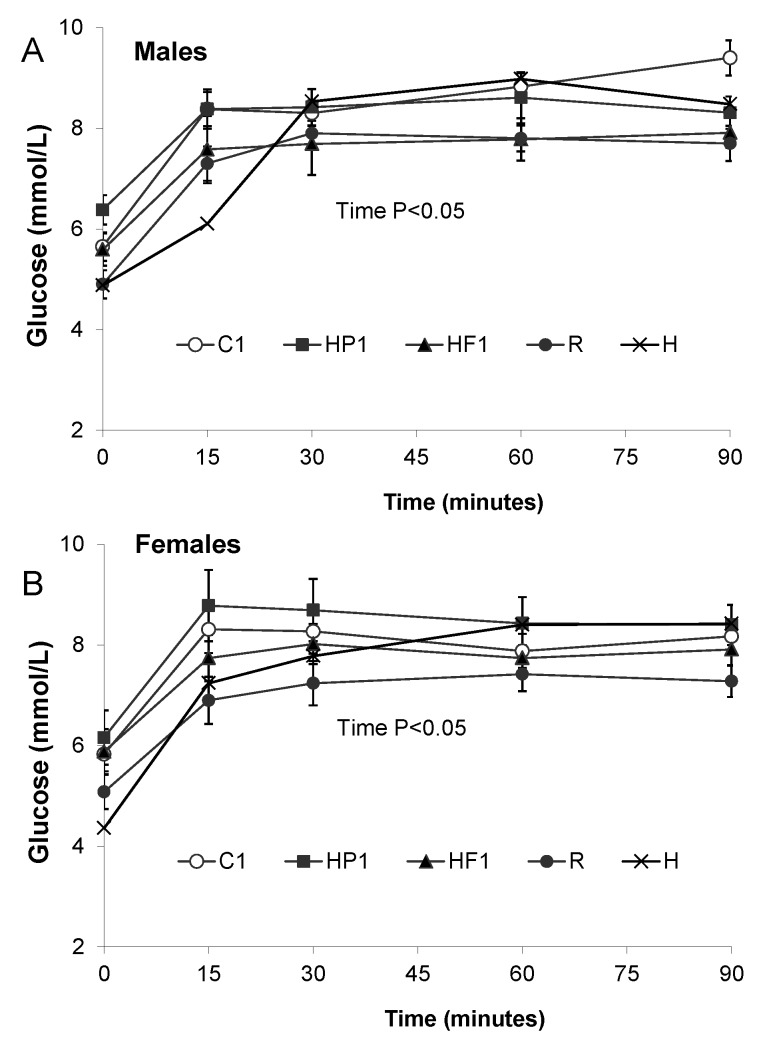
Blood glucose concentrations during an oral glucose tolerance test in male (**A**) and female (**B**) offspring. Values are means ± SEM, *n* = 10 per group except *n* = 4 for group H. There was a significant effect of time (*p* < 0.05) for males and females.

Glucose AUC was lower in HF1 than C1 animals at 28 weeks of age (*p* = 0.001) while AUC for insulin was lower in HF1 and HP1 compared to C1 (Figure 4). The interaction between diet and sex affected leptin AUC (*p* = 0.033) with male C1 rats having higher leptin than male HP1 and HF1 (Figure 4). AUC for the gut satiety hormones PYY and GLP-1 was higher in HF1 rats compared to C1 and HP1 (*p* < 0.001) (Figure 5). Conversely, HF1 rats had lower GIP AUC compared to C1 at 28 weeks of age (*p* = 0.026). Serial concentrations of glucose over the course of the OGTT are presented in Figure 6. There was a significant effect of time (*p* < 0.05) but no significant interaction effect between time and diet or sex during the course of the OGTT.

#### 3.1.4. Liver Triglyceride

Diet and sex independently affected liver triglyceride concentrations (*p* = 0.001). Liver triglycerides were higher in males than females, and lower in HF1 than C1 and HP1 rats at 28 weeks of age (Table 3).

#### 3.1.5. Hepatic Gene Expression

Diet had a significant effect on expression of hepatic SREBP-1c, ACC, FAS, and AMPKα1 (*p* < 0.03) with HP1 rats having higher SREBP1c mRNA levels than HF1 and C1; increased ACC and AMPKα1 mRNA levels than HF1; and increased FAS compared to C1. Sex had a significant effect on SREBP1c and FAS mRNA levels with males having higher expression than females (*p* < 0.03). The interaction of diet and sex affected PGC1α (*p* < 0.01) and glucose-6-phosphatase mRNA levels (*p* < 0.02) with higher levels in HP1 *versus* C1 and HF1 (Table 4). It should be noted that many of the genes measured have post-translational regulatory factors, resulting in potential differences between mRNA expression and physiological function.

**Table 4:** Hepatic gene expression in the offspring of dams fed a control, high-protein or high-prebiotic fiber diet during pregnancy and lactation and then re-matched to their respective diets in adulthood.

		Diet Group			*p* (2-Way ANOVA)		
	Sex	HP1	HF1	C1	Diet	Sex	Diet × Sex
SREBP-1c	M	5.5 ± 0.73	2.6 ± 0.7	4.6 ± 0.67	0.002	0.002	0.143
	F	4.0 ± 0.66	2.1 ± 0.47	1.7 ± 0.53			
ACC	M	13.3 ± 2.0	10.9 ± 2.0	15.0 ± 1.6	0.032	0.553	0.097
	F	18.9 ± 3.1	10.6 ± 1.2	12.6 ± 1.3			
FAS	M	6.8 ± 2.0	4.7 ± 1.1	4.0 ± 0.74	0.002	0.001	0.124
	F	18.2 ± 3.1	13.0 ± 1.9	8.2 ± 0.92			
PGC1α	M	88.9 ± 19.6 ^y^	45.9 ± 9.8 ^x^	24.1 ± 5.8 ^x^	0.001	0.064	0.972
	F	110.9 ± 21.1 ^y^	62.5 ± 13.6 ^x^	45.7 ± 6.4 ^x^			
Glucose-6-Phosphatase	M	23.6 ± 5.5 ^x^	19.5 ± 3.8 ^x,y^	17.1 ± 2.7 ^x,y^	0.001	0.031	0.014
	F	43.6 ± 4.4 ^y^	16.7 ± 2.9 ^x^	20.5 ± 2.3 ^x^			
AMPKα1	M	16.5 ± 1.6	11.7 ± .9	10.7 ± 1.4	0.006	0.587	0.121
	F	16.2 ± 2.8	9.7 ± 1.8	15.2 ± 1.0			

C1, offspring of control dams re-matched to control; HP1, offspring of high protein (HP) dams re-matched to HP; HF1, offspring of high prebiotic fibre (HF) dams re-matched to HF; SREBP1c, sterol regulatory element-binding protein-1c; M, male; F, female; ACC, acetyl-CoA carboxylase; FAS, fatty acid synthase; PGC1α, Peroxisome proliferator-activated receptor gamma coactivator-1-alpha; AMPKα1, AMP-activated protein kinase alpha-1. Values are means ± SEM, *n* = 10 per group except *n* = 4 for group H. Values with different letters are significantly different from each other (*p* < 0.05).

### 3.2. Effect of HFS Exposure and Reversibility

The following results describe the outcomes in offspring that were never exposed to the HFS diet and consumed the control AIN-93 diet throughout the study called the reference group (R); offspring continued on HFS for an additional 6 weeks post-HFS challenge (H); or re-matched to the control AIN-93 diet for 6 weeks following the HFS challenge (C1).

#### 3.2.1. Growth and Energy Intake

For the control rats that either continued on AIN-93 control diet (R), switched back to AIN-93 diet following HFS (C1) or continued on HFS (H), there was a significant effect of time but not diet on body weight (*p* < 0.001) (Figure 2). Energy intake at 24 weeks was greater in C1 than H (*p* = 0.025) (Figure 3). At 27 weeks, energy intake was greater in R than C1 and was also greater in males than females (*p* = 0.037, *p* < 0.001).

#### 3.2.2. Body Composition

There was no statistical difference in percent body fat at 28 weeks of age between the three groups of offspring from control dams; namely C1, R or H (Figure 3). Lean mass (*p* = 0.009) was lowest in C1 for males and females and BMD (*p* = 0.004) was lower in C1 males than R and H. Diet had a significant effect on organ weight measured at 28 weeks of age (Table 2). Stomach weight (*p* = 0.003) was lower in C1 while cecum weight (*p* < 0.001) was greater in R than C1 and H. There was also a significant sex effect for these parameters, with males having higher values than females (*p* < 0.001).

#### 3.2.3. Plasma Satiety Hormones and Blood Glucose

The interaction between diet and sex affected fasting amylin and insulin wherein amylin was higher in male H than C1 and R rats (*p* < 0.001), and fasting insulin was higher in male C1 than R and H rats (*p* = 0.006) (Table 3). Diet had a significant effect on fasting glucose (Table 3) and AUC for glucose, amylin and ghrelin (Figure 4 and Figure 5). C1 rats had higher fasting glucose than R and H rats (*p* = 0.001) and greater glucose AUC (*p* < 0.001) and amylin AUC (*p* < 0.001) than R rats. GLP-1, fasting and AUC, was higher in R than C1 females (*p* = 0.03) (Figure 5). Ghrelin AUC (*p* = 0.021) was greater in R than C1.

## 4. Discussion

Our first aim was to determine if the effects of a transient HFS diet could be attenuated by re-matching offspring in adulthood to the diet they were exposed to *in utero*. Re-matching to a diet high in prebiotic fiber (HF1) reduced body weight and percent body fat compared to both C1 and HP1. We previously reported that a maternal diet high in prebiotic fiber protected female offspring against diet-induced obesity from a HFS challenge, while a maternal diet high in protein pre-disposed female offspring to greater fat mass accumulation [8]. The protection is similar when the diet is introduced at weaning and rats that consume a high prebiotic diet from weaning to adulthood are more resistant to diet-induced obesity compared to control and high protein fed animals [7]. In the current study, the decreased body weight and adiposity in HF1 rats is likely due in part to increased levels of the satiety hormones GLP-1 and PYY. These hormones have frequently been reported to decrease food intake and increase feelings of fullness [14].

Insulin resistance, as measured by HOMA-IR scores, was lower in HF1 and HP1 males than C1 males. Previously, we reported that HOMA-IR scores were lower in HP1 animals than C1 at the end of the HFS period [8]. This protection appears to persist when the rats are re-matched to the HP diet. While a meta-analysis of the long-term effects of low *versus* high protein diets on metabolic risk factors in human clinical studies [15] did not identify any difference in fasting glucose or glycosylated hemoglobin, fasting insulin was significantly reduced with the high *versus* low protein diet which is consistent with our data in males. The lower HOMA-IR in the HF animals is consistent with previous reports of improved glycemic and insulinemic response with prebiotic intake [16].

HF1 animals had decreased liver triglyceride concentration compared to HP1, retaining the same pattern as previously reported following the HFS challenge [8]. HP1 animals in addition to having greater hepatic triglyceride levels also demonstrated increased gene expression of the lipogenic enzymes acetyl Co-A carboxylase (ACC), fatty acid synthase (FAS) and sterol regulatory element-binding protein-1c (SREBP1c) in the liver. These enzymes are integrally involved with *de novo* fat synthesis and have been shown to increase with certain dietary modifications [17]. For example, fatty liver induced by fructose feeding is associated with increased SREBP-1c and FAS mRNA levels and has been shown to be produced by both insulin-dependent and independent pathways [18,19]. Furthermore, high fat diets also increase the expression of ACC, FAS and SREBP-1c [20]. While studies evaluating the effect of high protein diets are more limited, there is evidence that supplemental protein in yaks increases body weight, intramuscular fat and expression of FAS, ACC and SREBP-1c [21].

It has previously been shown that supplementation of the prebiotic fructooligosaccharide results in enlargement of the cecum [22]. As the cecum is the location for fermentation of non-digestible components of the diet, increased fermentation will increase the size of the cecum [23]. We expected and confirmed that HF1 animals had increased cecal mass due to the fermentation of the prebiotic. One of the bacterial species increased with prebiotic fiber intake is *Bifidobacterium*. This species is strongly associated with gut health and has also been found to have an impact on whole body metabolic health [24]. Specifically, increases in *Bifidobacterium* spp. are associated with improved measures of glycemia and insulinemia [25]. Increases in bifidobacteria with the HF diet, which we have previously shown [26,27] are likely to explain some of the beneficial effects on glucose tolerance and gut satiety hormones seen when rats were re-matched to the HF diet.

Our second aim was to examine the effects of transient *versus* persistent exposure to a high fat, high sucrose diet in offspring whose mothers consumed a control diet. We compared a variety of metabolic outcomes in animals that were never exposed to HFS, exposed for the final 14 weeks and those that were re-matched to control diet following the 6 week HFS challenge. Perhaps not surprising given evidence that a diet high in fat and sugar will increase glycemia [28,29], the animals that were never exposed to HFS diet had lower fasting glucose and glucose AUC compared to those that transiently consumed the HFS diet for 8 weeks (*i.e.*, C1). Females in the R group who were exposed solely to control diet pre- and postnatally also had higher fasting GLP-1 and GLP-1 AUC than those with transient exposure to the HFS diet. GLP-1 is an incretin hormone with glucose-lowering properties, secreted from the l-cells of the distal small intestine and proximal colon [30]. This hormone has been shown to be negatively impacted by high fat diets wherein obesity-prone rats consuming a high fat diet have lower plasma GLP-1 levels and a decreased number of l-cells in the distal small intestine [31]; therefore higher GLP-1 levels in the R group would be expected.

In contrast to what one might expect with prolonged exposure to a HFS diet [28,29], animals that remained on HFS for the final 14 weeks of the study in fact had lower fasting glucose compared to the C1 animals in which the exposure to the HFS diet was transient. Insulin levels of animals switched from a cafeteria diet to chow have previously been reported to be three times higher than those never consuming a cafeteria diet [32]. Similarly, our C1 rats re-matched to the control diet after 8 weeks of HFS consumption had higher fasting insulin levels than reference R rats. However, contrary to results from South *et al.* [32] where any exposure to the cafeteria diet resulted in elevated insulin, our male rats maintained on the HFS diet did not have significantly higher fasting insulin levels than those never exposed to HFS. Fasting glucose was also highest in C1 animals and therefore reflective of HOMA-IR scores which point to greater insulin resistance with transient exposure *versus* no exposure or prolonged exposure to HFS diet. In a similar diet switch study, rats exposed transiently to a low protein diet showed higher insulin resistance, hyperglycemia and body fat compared to rats fed normal protein continuously, an observation linked to altered sympathetic and vagus tonus [33]. Our results suggest that transient exposure to HFS diet can impair glucose handling and this effect is still evident 6 weeks after switching back to a control diet.

Despite the differences observed in glucose tolerance, rats that were re-matched to control diet after the HFS challenge did not display any differences in body weight or body composition from rats that continued to consume HFS over the final 6 weeks re-matching period. This could be considered “persistent” obesity, as has been reported elsewhere and points to a persistent elevation of body weight and fat mass after the removal of obesogenic diets [34,35]. This is in contrast to other studies that have shown the reversibility of diet-induced obesity and reported weight loss following a switch from a high fat diet to a control chow diet [36,37]. Whether the persistence of obesity occurred in our animals is difficult to conclude due to the similar body weight and body composition to those rats never exposed to the HFS diet (*i.e.*, R rats). The control diet used in this study was AIN-93. Unpublished data from our laboratory has shown that percent body fat is higher in rats consuming the purified powdered AIN-93 diet compared to a standard pelleted chow diet and is not statistically different from fat mass in rats fed a HFS diet for 8 weeks (Neustadter and Reimer, unpublished results [38]). Rodent diet provided in powdered *versus* pellet form is easier for animals to eat and typically contains ingredients that make it more palatable, such as Dyetrose, a selectively depolymerized corn starch rich in tetrasaccharides. In a comparison of soft and hard food, Sako *et al.* [39] found that when given the choice, rats would consume more soft food than hard pellets. The lack of weight loss in our C1 animals compared to other studies that switch from a HFS to control diet [36,37] could be due to the palatability and softness of our powdered diet compared to chow pellets which likely impacted food intake and subsequent body weight. Indeed reference animals consumed more energy during the final food intake measurement period than C1 animals, neither of which was different from H animals. The similarity in palatability of our control AIN-93 diet and HFS diets may have played a role in the persistence of obesity given a previous report that decreased food intake and weight loss occurred only when there was a drastic difference in palatability of the diet from liquid Ensure to chow [40].

A limitation of this study design is the inability to distinguish between the lasting effects of maternal diet during pregnancy and maternal diet during suckling. As organ and metabolic systems are in different developmental phases in the offspring in the fetal and early postnatal periods, there is the potential for differential effects of maternal diet that are unique in each period and this should be examined further.

In conclusion, changes in diet composition across the lifespan are a common occurrence [41,42]. Evidence suggests that when the dietary mismatch across periods is great, the susceptibility to chronic disease increases [43]. This study suggests that re-matching offspring to a maternal diet high in prebiotic fiber has clear benefits for reducing the negative impact of a transient HFS diet, including reduced body weight and body fat and improved satiety hormone response in adulthood. Although re-matching offspring to a maternal high protein diet resulted in significantly higher body fat than HF1 rats, they were no worse than C1 animals and the males in fact had better HOMA-IR scores than C1. The surprising lack of difference in body fat between C1, R and H animals suggests that the obesogenic potential of the AIN-93 diet warrants further investigation.

## Abbreviations

The following abbreviations are used in this manuscript:

HFS: High fat/high sucrose
HF: High prebiotic fiber diet
HP: High protein diet
C: Control diet
R: Reference group that consumed control diet throughout
C1: Rats switched back to control diet after high fat/sucrose diet
HF1: Rats switched back to high fiber diet after high fat/sucrose diet
HP1: Rats switched back to high protein diet after high fat/sucrose diet
AIN-93: American Institute of Nutrition-93
GLP-1: Glucagon-like peptide 1
PYY: Peptide tyrosine tyrosine
GIP: Glucose-dependent insulinotropic polypeptide
OGTT: Oral glucose tolerance test
DXA: Dual-energy x-ray absorptiometry
HOMA-IR: Homeostasis model assessment of insulin resistance
ACC: Acetyl Co-A carboxylase
FAS: Fatty acid synthase
SREBP-1c: Sterol regulatory element binding protein-1c
AMPKα1: Adenosine monophosphate-activated protein kinase alpha-1
GAPDH: Glyceraldehyde-3-phosphate dehydrogenase
CT: Cycle threshold
AUC: Area under the curve
